# Efficacy of a training intervention on the quality of practitioners' decision support for patients deciding about place of care at the end of life: A randomized control trial: Study protocol

**DOI:** 10.1186/1472-684X-7-4

**Published:** 2008-04-30

**Authors:** Mary Ann Murray, Annette O'Connor, Dawn Stacey, Keith G Wilson

**Affiliations:** 1 Faculty of Health Sciences, School of Nursing, University of Ottawa, 451 Smyth Road, Ottawa, Ontario, K1H 8M5, Canada

## Abstract

**Background:**

Most people prefer home palliation but die in an institution. Some experience decisional conflict when weighing options regarding place of care. Clinicians can identify patients' decisional needs and provide decision support, yet generally lack skills and confidence in doing so. This study aims to determine whether the quality of clinicians' decision support can be improved with a brief, theory-based, skills-building intervention.

**Theory:**

The Ottawa Decision Support Framework (ODSF) guides an evidence based, practical approach to assist clinicians in providing high-quality decision support. The ODSF proposes that decisional needs [personal uncertainty, knowledge, values clarity, support, personal characteristics] strongly influence the quality of decisions patients make. Clinicians can improve decision quality by providing decision support to address decisional needs [clarify decisional needs, provide facts and probabilities, clarify values, support/guide deliberation, monitor/facilitate progress].

**Methods/Design:**

The efficacy of a brief education intervention will be assessed in a two-phase study. In phase one a focused needs assessment will be conducted with key informants. Phase two is a randomized control trial where clinicians will be randomly allocated to an intervention or control group. The intervention, informed by the needs assessment, knowledge transfer best practices and the ODSF, comprises an online tutorial; an interactive skills building workshop; a decision support protocol; performance feedback, and educational outreach. Participants will be assessed: a) at baseline (quality of decision support); b) after the tutorial (knowledge); and c) four weeks after the other interventions (quality of decision support, intention to incorporate decision support into practice and perceived usefulness of intervention components). Between group differences in the primary outcome (quality of decision support scores) will be analyzed using ANOVA.

**Discussion:**

Few studies have investigated the efficacy of an evidence-based, theory guided intervention aimed at assisting clinicians to strengthen their patient decision support skills. Expanding our understanding of how clinicians can best support palliative patients' decision-making will help to inform best practices in patient-centered palliative care. There is potential transferability of lessons learned to other care situations such as chronic condition management, advance directives and anticipatory care planning. Should the efficacy evaluation reveal clear improvements in the quality of decision support provided by clinicians who received the intervention, a larger scale implementation and effectiveness trial will be considered.

**Trial registration:**

This study is registered as NCT00614003

## Background

There are more choices for place of end-of-life cancer care due to shifts in care to the community, better understanding of the clinical course of cancers, equipment portability, pharmacology advances, and consumer expectations. Place of care has various meanings to patients and families and represents more than a particular geographic location [1]. Options can include hospice, private residence, nursing home, continuing complex care facility, homeless shelter, and/or hospital. For patients with good symptom control, instrumental support, and a predictable course of illness, decisions regarding place of care are often based on values and expectations [2-5].

Patients frequently experience decisional conflict (personal uncertainty about the best course of action) when considering place for end-of-life care. Personal preferences are weighed against practical considerations and concern for others [2,3,5,6], thus contributing to decisional conflict. Other modifiable factors such as knowledge gaps, unrealistic expectations about outcomes, lack of clarity about what matters most, and feeling pressured to choose a particular option exacerbates the decisional conflict [7]. Unresolved decisional conflict can lead to decisional delay or reversal, dissatisfaction, regret, and blaming the provider [8,9]. Failure to elicit the valued priorities of terminally ill patients and their families can result in missed opportunities, decreased quality of life, unwelcome interventions, and increased risk for complicated bereavement for survivors [3,10-12].

It is generally known that decisional conflict can be reduced with decision support interventions such as decision aids and nurse coaching [7,13]. However, there have been few studies evaluating either of these interventions for decisions at the end-of-life. Although patient decision aids may be useful for some common discrete crossroads decisions with standardized options and outcomes, there may be more payoffs in focusing on coaching interventions that can be applied broadly to care management at the end-of-life. While end-of-life decisions are highly values-sensitive, they also bear a strong resemblance to chronic condition management decisions, which focus on situation monitoring, priority setting, and implementation [14-16].

There is evidence that influencing practitioners' knowledge and attitudes about communication and decision support can strengthen subsequent decision support practices, thus matching care planning to patient preferences and avoiding the use of non-valued interventions [17-20]. Nevertheless few studies have empirically examined the impact of a theoretically informed, decision support training intervention on the quality of decision support knowledge and practices provided by practitioners [21] and none have been undertaken within the context of palliative care practice.

Patients and families want clinicians to listen to their views and preferences [18] and standard palliative practice calls for patient inclusion in care planning [22,23]. Most patients with advanced cancer want full information and the majority wish to participate actively in decision making [24]. Although seriously ill hospital patients want to discuss end-of-life issues, their preferred decision making role varies and is difficult to predict [25], highlighting the need for active and regular assessment of patients' decision making needs. However, clinicians may avoid raising uncomfortable topics [26-28] and often lack skills and confidence in helping patients in non-directive ways [29].

Systematic review findings confirm that training clinicians in patient centered approaches is an effective strategy for increasing patients understanding of the evidence and implications [30]. For instance, decision coaching by nurses has helped to foster an informed use of resources and avoid the over-use of interventions that patients don't value in urology care [17] and in gynecological care [31]. Practitioners, such as nurses and other professional care coordinators, through their trusted and frequent interactions with patients are well positioned to elicit and explore decisional needs such as decisional conflict and related factors (e.g.: knowledge, values clarity, and support) [32]. Practitioners can then coach patients with information, values clarification, support and links to resources. While a key challenge is the need to strengthen practitioners' decision support skills [29] training interventions have been shown to markedly improve the quality of practitioners' decision support skills for other clinical problems [33].

Accountability to quality patient outcomes and fiscal responsibility confirms the need to practically and pragmatically address patients' end-of-life decision making needs. Systematic and rigorous evaluation of an evidence-based intervention designed to improve the quality of practitioners' decision support could illuminate best practices for decision support and advance the fields of shared decision making, patient-practitioner communication, palliative care, and ultimately improve the lives of those who are living with a terminal illness and their families.

Multifaceted interventions show promise in influencing professional behavior change [34-37]. An exploratory study to identify target variables, choose and refine interventions, and establish their theoretical basis prior to large scale effectiveness trials is a sound research approach [38-40]. The paucity of empirical inquiry, in this area to date, warrants an exploratory study to describe the constant and variable components of a potential intervention and a feasible protocol for intervention delivery.

### Guiding Theoretical Models

As the study aims to influence decision support behavior an understanding of factors that can be partially modified, such as attitudes and perceptions of norms that drive actions, is required to inform intervention messages and enhance the potential for behavior change. The pragmatic and conceptual focus of the study also requires an empirically proven, clinically relevant decision support framework to guide intervention content. The Theory of Planned Behavior (TPB) [41,42] and the Ottawa Decision Support Framework (ODSF) [43] fit these criteria. The former predicts the likelihood of behavior change while the latter provides a three step path to optimize quality decision support. The TBP [40,44-46] and the ODSF [3,47,48] are relevant to nursing and have performed well in numerous health-related studies.

Briefly, the TPB proposes that the strength of the intention to change is the primary determinant of actual behavior change. This intention is determined by: (1) a person's attitude to the new behavior (strength of perceived advantages and disadvantages); (2) the extent of self-perceived social pressure to perform or not to perform the new behavior, and (3) degree of perceived control over being able to perform the behavior [49].

According to the ODSF, decision support interventions can be tailored to address modifiable determinants of decisional conflict (knowledge; outcome expectations; values clarity ; support factors) resulting in better quality decisions that are informed and consistent with patients' values [50]. In this study, the ODSF will guide decision support skill acquisition interventions and measures of change.

Clinician-identified factors associated with practice change: utility, strong evidence basis, and flexibility to acknowledge the individuality of patients [51] fit well with the ODSF. Historically, the ODSF has been well received by clinicians [3,33,48], has shown robustness in randomized control studies [7,33] and is predicated on a patient-centered approach which recognizes the unique context, circumstance and patient characteristics situated within the decision support encounter. The TPB and ODSF have guided the study design in the following ways:

1. The predictive potential of theory facilitates selection of training intervention components which offers the best probability of success [36,52].

2. The TPB offers a useful lens to examine attitudes, beliefs and perceived control for engaging in decision support practices and will inform the selection of key messages attached to intervention strategies.

3. Mapping the intervention components onto TPB variables provides a useful template to ensure multiple targets of behavior change are addressed and is consistent with knowledge dissemination best practices [35,53].

4. The ODSF describes a well defined approach to quality decision support provision and provides a practical vehicle to structure components of a knowledge and skill building intervention.

5. Availability of a broad inventory of theoretically grounded and empirically validated, reliable, time tested tools operationalizing ODSF constructs provides a rigorous platform to inform study interventions and will strengthen the trustworthiness of study findings.

## Methods/Design

### Aims of the study

The primary aim of this study is to evaluate the efficacy of a theory driven, framework-based training intervention, as compared with a control condition (usual care approach) in enhancing the quality of practitioners' patient decision support skills regarding place of care at end of life. In addition, we plan to assess participants' intention to engage in patient decision support in their practice and to determine the acceptability of the intervention components. Specific objectives include:

1. To identify factors affecting the likelihood of practitioners' integrating decision support principles into their practice

2. To determine the quality of decision support practitioners provide

3. To design and evaluate components of a decision support training intervention

We plan to test the following study hypothesis:

**H_1_**: A significantly greater proportion of practicioners, who are randomized to a multi-faceted, theory driven, training intervention, will obtain higher scores on quality of decision support following the intervention

**H_0_**: No change in group means in decision quality following the intervention

#### Study Design and Methods

A two phase, sequential, mixed method design is planned.

**Phase 1 **involves a focused needs assessment of key informants (clinicians, educators, administrators) who plan and provide palliative/oncology care. A purposive sample of about 12 key informants representing different levels of experience, responsibility, clinical focus and setting will be interviewed using a semi-structured interview based on the TBP [54].

**Phase 2 **is a randomized control trial of a brief educational intervention. Consenting/eligible participants will be randomly allocated to an intervention or control group. The intervention, informed by the needs assessment, comprises: an online tutorial; an interactive skills building workshop, a decision support protocol; performance feedback, and educational outreach.

Participants will be assessed: a) at baseline (quality of decision support); b) after the tutorial (knowledge); and 3–6 weeks after the other interventions (quality of decision support; intention to adopt decision support into clinical practice).

#### Sample Size

The estimated sample size for the Phase 2 study is based on a test for differences in mean scores of decision support quality and knowledge in the intervention versus the control group. An effect size of .70 requires n = 32/group, when alpha error = 0.05 and beta error = 0.20 [55]. This effect size is conservative in that a previous study (17) reported larger effect sizes which required only 18–20 per group.

#### Participants

Full or part time nurses and care coordinators (i.e.: social worker, case managers, pharmacists) from three Ontario regions (Ottawa, Toronto, Kingston), employed by one of the ten study partner organizations will be invited to participate. Study partners include hospital based institutions [Ottawa Hospital, Queensway-Carleton Hospital, SCO Health Services], community based organizations [Community Care Access Center Care (Toronto Central; Southeast Ontario); CareFor Nursing Agency; St. Elizabeth Health Care (Toronto, Kingston); Hospice at May Court; Bayshore Home Health Agency (Cornwall, Toronto, Ottawa)]. Information flyers and information sessions explaining the study will be held at study partner organizations. Interested potential participants will be asked to contact the study coordinator if they have further questions or would like to participate in the study. Participants are considered eligible for study inclusion when they meet the following criteria:

• are a member of a regulated health profession

• care for palliative cancer patients, and/or

• cancer patients with advanced disease, and

• work at least 4 shifts per month,

• in a clinical area where end of life care discussions are likely to be undertaken, and

• are proficient in written and spoken English.

#### Ethical considerations

The protocol has been approved by the Research Ethics Committees of the University of Ottawa, The Ottawa Hospital and SCO Health Services. Trial registration with the International Standard Randomized Controlled Registry has been obtained (Trial #NCT00614003).

#### Procedures

##### Phase 1: Needs Assessment

Semi-structured interviews with a purposive sample of key informants (n ≅ 12 engaged in direct care, education and/or administration roles) will be used to elicit TPB related factors affecting intentions to provide decision support (personal attitudes, norms, and perceived control). Questions about other barriers to providing decision support will be elicited. Results will inform intervention content and key messages.

##### Phase 2 Intervention

Consenting practitioners will be randomly allocated to one of two groups. A computer generated randomization list for concealed allocation will be used. To avoid disparate sample sizes permuted blocks will be used. Participants will be allocated by an external statistician, who has no connection to the study team, immediately after collection of baseline data.

##### Baseline measures of both groups

The quality of decision support skills will be assessed using audio-taped interactions between participants and simulated patients. Simulation scenarios have been created and vetted by a panel of Palliative Advanced Practice Nurses. Simulated patient callers will receive a training and feedback session facilitated by an experienced trainer prior to placing the call to participants. Simulated patient callers will contact each participant and engage in a standardized scenario expressing difficulty related to a place of end-of-life care decision. Calls will be tape recorded and quality scored using the Decision Support Analysis Tool (DSAT) [56].

##### 2. Intervention (experimental) group

Participants assigned to the intervention group will:

a. Complete an on-line decision support tutorial which introduces the 'When you need extra care decision aid', case studies and quizzes related to place of care decision support.

b. Participate in a half day skills building workshop. The workshop contains aggregated feedback on baseline simulated calls; opportunities to observe, compare and contrast a traditional patient education approach to a patient decision support approach; role play using the 'When you need extra care decision aid' and peer scoring of the quality of their decision support provision using the DSAT. A determinants of place of care knowledge module will also be delivered. At the end of the workshop, participants will complete a questionnaire eliciting perceived utility/usability of the a) workshop content, and b) decision support protocol.

c. Participate via telephone in an education outreach session to identify areas needing clarification, share problem solving in using the decision support protocol, and to obtain further feedback.

d. Complete a questionnaire eliciting satisfaction with content and process of education program.

##### 3. Post measures of both groups

a. After the auto-tutorial, knowledge tests will be administered.

b. 2–6 weeks following completion of the full intervention a TPB based survey eliciting behavioral intention to integrate decision support in practice and related attitudes, norms, and perceived control will be administered.

c. 2–6 weeks following completion of the full intervention the quality of the participants' decision support skills will be assessed with a different simulated patient scenario using the same methodology as the baseline assessment.

**Proposed Intervention **Details of the educational intervention follow.

#### Decision Support Tutorial

Developed by Ottawa Health Decision Centre, Clinical Epidemiology Unit of the Ottawa Health Research Institute (OHRI) this on-line, self-learning resource has been used to train clinicians, graduate students and undergraduate nursing students; US and BC tele-health center nurses; UK urology nurses; and nurses in family practice units in Ontario. Three modules provide 1) an overview of decisional conflict with a three step path to guide decision support, 2) case studies profiling decision support tools (including a decision support protocol) and processes with embedded quizzes to assess comprehension and provide feedback; and 3) a final integrated knowledge test. The original tutorial will be adapted to decisions about the place of terminal care. The tutorial is hosted by the University of Ottawa and is password protected. Participants will be asked to complete the auto-tutorial one week prior to the workshop.

#### Decision Support Protocol

Participants will be introduced to a decision aid in the tutorial. The decision aid guides patients in advanced planning of location of care and is entitled 'When you need extra care decision aid'. The decision aid is based on the Ottawa Decision Support Framework and the Ottawa Decision Support Practitioners Guide (OPDG). The four page decision aid provides a structured approach to assess patients' decisional needs, provide tailored decision support to address needs and evaluate patients' progress in decision making. There are five elements: 1) general information about place of care options and palliative care; 2) self report of functional and symptom status over the past week based on the Palliative Performance Scale [57] and the Edmonton Symptom Assessment Scale [58] respectively; 3) a self-ranking of which reasons for each option are considered most important; 4) an assessment of what else patients need to prepare for decision making; and 5) a summary of next steps.

Content is based on: a) systematic reviews of literature [4,59,60]; b) previous research on women's decision making needs regarding place of care at the end of life [3]; previous research on family members' decision making needs at the end of life [61] and d) the primary investigator's clinical experience in palliative care. Participants will be provided with a hard copy of the protocol as well as access to the online version.

#### Skill building workshop

Within three weeks of the online tutorial, a half day workshop will be conducted. Content will be based on the pre-intervention TPB needs assessment and also a) practical applications of material learned in the tutorial; b) a video illustrating a clinical application of the decision aid; c) a video contrasting a traditional patient education approach and a decision support approach in a clinical scenario d) role play using the decision support protocol; e) self and peer appraisal during role play and f) discussion about barriers and facilitators to integrating decision support into clinical practice. Use of a facilitator who provides face to face communication and uses a range of enabling techniques has been shown to have some impact on changing clinical practice [62].

Specific workshop objectives are that participants will:

• understand concepts of decisional needs, decision support, and decision quality,

• learn to use decision support tools,

• evaluate decision support skills, and

• analyze barriers and facilitators to implementing decision support in practice.

#### Performance feedback

Results of the decision quality scored transcripts of baseline simulated calls will be presented and discussed at the workshop. Participants will also be provided with evaluation tools based on the Decision Support Analysis Tool (DSAT) to self appraise their own and workshop peers' quality of decision support during the case studies and role play activities. As well, the facilitator will provide ongoing feedback from case studies and role plays during the workshop. The DSAT self-appraisal tool has been used to train nurses and medical residents in self-appraisal at the University of Ottawa, the Dartmouth Hitchcock Medical Center, and the US Health Dialogue call center.

#### Education outreach

Two weeks following the workshop intervention group participants will be scheduled for a personal academic detailing session with the workshop facilitator. Based on social marketing approaches educational outreach provides a focused opportunity to personalize learning and behavioral objectives, provide unbiased descriptions of research evidence and opinion leaders' positions, augment educational materials and reinforce positive behavior [63]. Academic detailing using brief, face-to-face interactions has shown promise for modifying physician and dentists practices [64,65] although one study reported initial resistance to the approach [66] and it had no effect as a single intervention [67].

The detailer will provide individualized information and resources, reinforce decision support behaviors, and help participants to identify opportunities for incorporating decision support behaviors into their practice. The one-to-one session will be scheduled at a mutually agreed time, will be conducted by telephone and should last about 15–30 minutes.

### Outcome Measures

#### Primary Outcome Phase 2

*Quality of Decision Support Skills *will be measured with DSAT modelled on the ODSF and Ivey's Problem Solving Model [68]. The DSAT, with total possible score of 12, assesses the quality of decision support and consists of subscales measuring decision support and communication in a practitioner/patient dyad. The tool demonstrated adequate inter-rater reliability for scoring on both decision support skills (75%, kappa = 0.58) and communication skills (76%, Kappa = 0.68) when it was tested in physician/patient dyads (n = 34 dyads). Construct validity was demonstrated when the scores were correlated to measures of patient and physician satisfaction[56] The DSAT also discriminates between trained and untrained nurses [33].

#### Secondary Outcomes Phase 2

Measures include:

• a knowledge test regarding decision support concepts;

• self assessment of decision protocol utility and helpfulness;

• behavioral intention to integrate decision support into clinical practice; and

• acceptability and utility of intervention components in the experimental group.

### Analysis Plan

#### Primary Outcome Phase 2: Quality of nurses' decision support skills

1. Inter-rater reliability of DSAT scores; two raters, who are blind to group allocation, will independently score pre and post intervention simulated call audio tapes.

2. Primary analysis will be undertaken using a repeated measures ANOVA (baseline; post measures). For missing cases an 'intention to treat', approach will be used under the conservative assumption that no change would occur between pre and post testing. Additionally, the impact of missing cases on findings will be explored between groups to provide further direction for the analysis.

#### Secondary outcomes Phase 2

1. Descriptive measures (frequency; means; range) will describe participant characteristics and acceptability and utility of training intervention components.

2. Descriptive measures (frequency; means; range) of the TPB based survey eliciting behavioral intention to integrate decision support in practice and related attitudes, norms, and perceived control will be undertaken. Between group differences in intention to integrate decision support practices will be analyzed using a t test.

3. Data from qualitative open-ended questions using traditional content analysis techniques [69,70] with the TPB as an organizing framework will be undertaken. Thematic coding, followed by member checking to ensure trustworthiness of final themes, will be undertaken.

## Discussion

This will be the first study to evaluate the impact of an educational intervention to improve the quality of decision support that practitioners provide to dying patients around place of care. This reproducible, portable intervention addresses a key policy mandate regarding choice for end of life care set out by health providers such as the Ontario Ministry of Health in Canada. This is a pragmatic trial with relatively inclusive entry criteria and we anticipate recruiting participants from across a spectrum of care sectors. As well, because we are bringing the intervention to participants in their home regions we are able to include participants who may be unable to access centrally held education due to time and distance pressures in their clinical setting. These features will improve the generalizability of the findings.

Expanding our understanding of how practitioners can best support palliative patients' decision making will help to improve the quality of end-of-life and care for patients and those who share their lives. If findings from this study show promise, a larger effectiveness trial assessing factors such as cost effectiveness, sustainability, and patient and system outcomes will be undertaken

Results will be disseminated via a brief summary prepared for policy makers, a communication flyer for participants, a technical report for the participating organizations, publication in refereed scientific journals, newsletters of palliative care and relevant clinician associations, presentations at scientific and clinical meetings, and clinical rounds. Findings will be available online through the websites of the Canadian Virtual Hospice, Canadian Institutes of Health Research (CIHR) Family Caregiving New Emerging Team, CIHR End-of-Life Care for Seniors New Emerging Team, and the CIHR/NCIC Strategic Training Program in Palliative Care Research.

### Design Limitations

Using a similar design in a call center nurses project, contamination was prevented using the following strategies: a) using a private room for simulated calls; and b) requesting that nurses not share or discuss decision support resources or approaches with others. Many of the skills are quite novel (values clarification) and it is unlikely that skills will improve without the workshop and subsequent practice.

Recruitment response may yield an over representation of those more motivated to learn and adopt the intervention than the average adopter. However, this should not pose a threat to internal validity. Moreover, involving early adopters is considered a wise strategy in innovation diffusion [71].

Participants will not be not blinded to the simulated call and know their performance is being monitored. However the use of simulated callers is recognized as a relatively reliable method for assessing professional performance, facilitates a standardized experience across participants, provides a clearer picture of decision support skills in general, is a more accurate measure of current practice compared to self-report or chart audit, and has been used widely [72-77].

The relative impact of each component of the intervention cannot be established with this design [40]. Feasibility constraints preclude a study design using a sequenced addition and evaluation of intervention components or assessing long term sustainability; however, recent studies suggest that evidence based education strategies may trigger long term practice change [78,79].

## Competing interests

The authors declare that they have no competing interests.

## Authors' contributions

MAM is the initiator of the study and drafted the manuscript. AO'C contributed significantly to the design of the study. AO'C, DS and KGW are providing supervision and mentorship during the implementation of the study and analysis of study data and have contributed to manuscript preparation. All authors read and approved the final manuscript.

## Pre-publication history

The pre-publication history for this paper can be accessed here:


